# Maternal chitosan oligosaccharide supplementation during late gestation and lactation optimizes placental function in sows and intestinal function in 21-day-old IUGR suckling piglets

**DOI:** 10.3389/fvets.2024.1463707

**Published:** 2024-11-13

**Authors:** Xintao Wang, Tingting Fang, Daiwen Chen, Junning Pu, Gang Tian, Jun He, Ping Zheng, Xiangbing Mao, Aimin Wu, Bing Yu

**Affiliations:** ^1^Animal Nutrition Institute, Sichuan Agricultural University, Chengdu, China; ^2^Key Laboratory of Animal Disease-Resistance Nutrition, China Ministry of Education, China Ministry of Agriculture and Rural Affairs, Key Laboratory of Sichuan Province, Chengdu, China

**Keywords:** chitosan oligosaccharide, intrauterine growth retardation, sows, piglets, placenta, intestine

## Abstract

Maternal dietary supplementation with chitosan oligosaccharide (COS) has been considered as a potential intervention to mitigate the occurrence of intrauterine growth restriction (IUGR) and improve postnatal growth. The present study investigated the effect of COS as a dietary supplement for sows during late gestation and lactation on their productivity, placental function, and the intestinal health of IUGR piglets. From day (d) 85 of late gestation to d 21 of lactation, 30 sows were randomly divided into either a control group (basal diet) or a COS group (basal diet + 100 mg kg^−1^ COS). At d 21 of lactation, eight normal and eight IUGR littermates from eight litters belong to control sows, as well as eight IUGR littermates from COS sows, were selected for further analysis. The results showed a significant reduction in the number of stillbirths and mummies in COS groups (*p* < 0.05). Maternal dietary supplementation with COS also significantly up-regulated the expression levels of *GLUT1*, *GLUT3*, and *VEGFA* mRNA in the placenta of IUGR piglets compared to those in control group (*p* < 0.05). Furthermore, there was a significant decrease in MDA content and a significant increase in GSH content in the placenta of IUGR piglets from COS sows compared to those from control group (*p* < 0.05). Additionally, the expression levels of *MUC2* and *occludin* mRNA as well as claudin1 protein significantly up-regulated in the jejunum of 21-day-old IUGR piglets from COS sows group compared to those from control group (*p* < 0.05). Moreover, *IL-10* mRNA expression level was significantly increased while MDA content was significantly reduced in the jejunum of 21-day-old IUGR piglets from COS sows group compared to those from control group (*p* < 0.05). The results indicated that maternal dietary COS supplementation during late gestation effectively reduced the incidence of stillbirths and mummies, potentially linked to enhanced placental function, reduced oxidative stress, and improved immune status. Furthermore, maternal dietary COS supplementation exhibited positive impact on intestinal digestive and absorptive function, intestinal barrier integrity, intestinal antioxidant capacity and immune status in 21-day-old suckling IUGR piglets.

## 1 Introduction

Intrauterine growth retardation (IUGR) in mammals refers to abnormal fetal growth and organ development in the uterus, resulting in low birth weight and impaired growth and development (1). Various factors, including maternal, fetal, placental or genetic influences, contribute to the formation of IUGR (2). In multiparous animals, we considered neonates whose birth weights did not align with the mean litter weight distribution and were less than twice the standard deviation as IUGR animals (3). The placenta plays a crucial role as a mediator between the mother and fetus. Therefore, oxidative stress, poor developmental status, and impaired nutrient transport of the placenta can collectively contribute to the occurrence of IUGR (4). IUGR adversely impacts neonatal morbidity and mortality, leading to impaired nutrient absorption and abnormalities in body composition, such as reduced muscle mass (5, 6). IUGR has become an issue that cannot be ignored in both nutritional human obstetrics as well as animal production. Recent studies have shown that nutritional interventions in sows can effectively reduce the incidence of IUGR. For instance, Pieszka et al. (7) showed the effectiveness of supplementing sow diets with pancreatic-like enzymes, while Wu et al. (8) found that supplementation with adenosine was also effective in reducing the occurrence of IUGR. However, current IUGR research primarily emphasizes direct maternal effects on the fetus and the characterization of the IUGR placenta. Our aim was to connect the mother, fetus, and placenta, and investigate how IUGR affects the offspring’s gut health.

Chitosan oligosaccharide (COS) is a degradation product of chitosan, typically prepared using physical, chemical, enzymatic, and other methods. COS has been demonstrated to have biological effects such as antioxidant (9), anti-inflammatory (10), antibacterial (11, 12), and anti-tumor activity (13). Study has proved that supplementing COS in the diet of pregnant sows can enhance sows health status, provide an improved growth environment for fetuses and increase the survival rate of newborn piglets (14). Additionally, supplementing sow feed with COS enhanced the antioxidant and anti-inflammatory abilities as well as the nutritional composition of sow milk, thereby improving the growth performance of piglets (15, 16). Furthermore, study has also indicated that COS improves amino acid transport capacity in the placenta and promotes nutrient transport from sow to fetuses (17). Existing studies focusing on reproductive performance (14), milk composition (18), antioxidant capacity (17), and placental capacity of sow with supplementation of COS to their diets have rarely investigated the effects of feeding COS to sows on IUGR piglets.

Therefore, we hypothesized that maternal COS supplementation during late gestation and lactation can improve placental function, reduce the incidence of IUGR, and improve the intestinal health of IUGR piglets. Thus, the aim of this study was to evaluate the effects of maternal supplementation with COS during late gestation and lactation on reproductive performance, placenta function, immune response, and antioxidant capacity of placenta in sows. Furthermore, digestive enzyme activity, intestinal barrier function, cytokine levels and antioxidant indicators were evaluated in piglet jejunum.

## 2 Materials and methods

### 2.1 Animal ethics

This experiment was performed in accordance with the Laboratory Animal-Guideline for ethical review of animal welfare of the People’s Republic of China (GB/T 35892-2018) and approved by Sichuan Agricultural University Animal Care and Use Committee.

### 2.2 Chitosan oligosaccharide

The effective content of COS used in this study is 10% with Maltose dextrin as the carrier, MW <1,000 Da, and degree of deacetylation (DD) is about 95%, which was provided by Zhongke Runxin (Suzhou) Biological Technology Co., Ltd. The amount of COS added was calculated based on the effective content.

### 2.3 Animals, diets and experimental design

This study was conducted in Zitong Original Breeding Pig Farm, which is a member of Sichuan Mianyang Jinchiyang Agriculture and Animal Husbandry Co., Ltd. A total of 30 sows (Landrace × Yorkshire) with specific criteria for back fat, parity, and expected delivery date close to 85 days of pregnancy, were randomly allocated to 2 groups: control group (control, sows fed basal diet) and COS group (COS, sows fed basal diet + 100 mg kg^−1^ COS) (14). The basal diets were formulated to meet the nutrient requirements of gestating sows or lactation sows, respectively, as recommended by the National Research Council (NRC, 2012), and their compositions and nutrients levels were shown in Tables 1, 2.

**Table 1:** Composition and nutrient levels of the basal diet for sow during late gestation.

Ingredient	Content, %	Nutrient levels^b^	Content
Corn	13.00	CP, %	16.5
Soybean meal (≥43%)	5.50	DE, Mcal/kg	3.00
Wheat	30.00	Ca, %	0.85
Rice bran	10.00	Available P, %	0.28
Puffed flaxseed powder	3.00	D-Lys	0.67
Beet pulp	4.00		
*Saccharomyces cerevisiae* ferments distiller’s grains	9.70		
Soybean hulls	6.00		
Bran (wheat bran)	8.60		
Fermented soybean meal	4.00		
Fish meal	1.00		
Expanded soybean	2.00		
Sodium chloride	0.37		
Calcium carbonate	1.11		
Dicalcium phosphate	0.82		
Sodium bicarbonate	0.10		
Acidifier	0.15		
Choline chloride	0.25		
Phytase	0.02		
Feed compound enzyme	0.03		
DL-α-tocopherol acetate	0.02		
Potassium chloride	0.03		
Mineral and vitamin premix^a^	0.30		
Total	100		

a Premix supplied the following per kg of diet: copper (CuSO_4_), 10 mg; iron (FeSO₄), 80 mg; zinc (ZnO), 50 mg; manganese (MnSO_4_), 25 mg; selenium (Na₂SeO_3_), 0.15 mg; iodine (KIO_3_), 0.14 mg; vitamin A, 4,000 IU; vitamin D_3_, 800 IU; vitamin E, 441 IU; menadione, 0.6 mg; thiamine, 1.0 mg; riboflavin, 3.35 mg; vitamin B_6_, 1.0 mg; vitamin B_12_, 15 μg; niacin, 10 mg; pantothenic acid, 12 mg; folic acid, 1.3 mg; D-biotin, 200 μg.

b Nutrient levels were calculated values.

**Table 2:** Composition and nutrient levels of the basal diet for sow during lactation.

Ingredient	Content, %	Nutrient levels^b^	Content
Corn	47.00	CP, %	17.00
Soybean meal	15.10	DE, Mcal/kg	3.32
Beet pulp	1.00	Ca, %	1.00
Wheat flour	8.00	Available P, %	0.35
*Saccharomyces cerevisiae* ferments distiller’s grains	3.00	D-Lys	1.10
Soybean hulls	1.50		
Bran (wheat bran)	5.00		
Fish meal	2.00		
Expanded soybean	2.00		
Five corn peptide	8.00		
Sucrose	2.00		
Soybean oil	1.00		
Sodium chloride	0.40		
Calcium carbonate	1.03		
Dicalcium phosphate	1.46		
Acidifier	0.30		
Choline chloride	0.15		
L-lysine HCL	0.52		
DL-methionine	0.03		
L-threonine	0.13		
L-tryptophan	0.02		
Phytase	0.02		
DL-α-tocopherol acetate	0.02		
Mineral and vitamin premix^a^	0.32		
Total	100		

a Premix supplied the following per kg of diet: copper (CuSO_4_), 10 mg; iron (FeSO₄), 80 mg; zinc (ZnO), 50 mg; manganese (MnSO_4_), 25 mg; selenium (Na₂SeO_3_), 0.15 mg; iodine (KIO_3_), 0.14 mg; vitamin A, 4,000 IU; vitamin D_3_, 800 IU; vitamin E, 441 IU; menadione, 0.5 mg; thiamine, 1.0 mg; riboflavin, 3.16 mg; vitamin B_6_, 0.90 mg; vitamin B_12_, 12.5 μg; niacin, 7 mg; pantothenic acid, 11 mg; folic acid, 1.3 mg; D-biotin, 180 μg.

b Nutrient levels were calculated values.

After delivery, the piglets were fostered to other gilts within the same treatment group to ensure each litter included 10–11 piglets, and the number of sows in both COS group and control group was adjusted to 10.

The piglets were breastfed until weaning at day 21. Piglets with a birth weight below 0.9 kg were classified as IUGR piglet. Newborn piglets categorized as NBW weigh 1.50 kg on average, while those categorized as IUGR weigh 0.84 kg. By day 21, their weights have increased to 6.00 kg and 3.17 kg, respectively.

### 2.4 Sample collection

Based on the birth weight of piglets, the placenta was classified into two categories: those corresponding to normal weight piglets and those corresponding to IUGR piglets. Following the onset of sow delivery, before cutting the umbilical cord as it emerged from the birth canal, three different colored silk threads (white for normal body weight piglet’s placenta in control group, NBW; pink for IUGR piglet’s placenta in control group, IUGR); and (black for IUGR piglet’s placenta in COS group, IUGR + COS) were used to tie off the respective umbilical cords of varying weight piglets. The marked umbilical cord with the silk threads were then retracted back into the sow’s body (the white and pink silk threads are of different materials, making it easier to distinguish them during sample collection). Once all placentas had been discharged by the sows post-delivery, fresh placental samples were collected from a 5 cm distance near the umbilical cord based on their color markings and stored in frozen tubes for subsequent testing after freezing in liquid nitrogen.

When piglets reach 21-day-old, those were anesthetized and euthanized, followed by a rapid opening of the abdominal cavity. The small intestine was then dissected, and tissue samples from the jejunum were promptly frozen in liquid nitrogen and stored at −80°C for subsequent analysis.

### 2.5 Sow reproduction

Record the changes in backfat of sows at days 85 and 111 of gestation, and day 21 of lactation, as well as the average litter size, live litter size, stillbirth, mummy size and the number of piglets with a birth weight below 1 kg. Calculate the average birth weight, live litter weight, percentage of IUGR, weaned litter size and weight, average weaned weight and daily gain during lactation for piglet, also calculate daily feed intake and backfat loss during lactation for sow. The percentage of IUGR refers to the proportion of piglets with BW <1.0 kg among the total born. Backfat loss was defined as the difference in backfat thickness between farrowing and weaning for each sow.

### 2.6 Antioxidant status of placenta in sows and jejunum in piglets

The levels of ATP, MDA, glutathione (GSH), and oxidized glutathione (GSSG) in the placenta, as well as the content of MDA, GSH, and GSSG and the activity of CAT, T-AOC, GSH-PX in the jejunum of piglet were determined using the commercial kits provided by Nanjing Jiancheng Bioengineering Institute (Nanjing, China). The total protein content in both the placenta and the piglet’s jejunum was measured using the same kit.

### 2.7 Placental cytokines concentrations

The levels of placental cytokines IL-1β, IL-10 and TNF-α were quantified using a swine ELISA kit (Beijing 4A Biotech Co., Ltd).

### 2.8 Activity of digestive enzyme in jejunum of piglets

The activities of trypsin, lipase, maltase, sucrase and lactase in piglet jejunum were determined using the commercial kits provided by Nanjing Jiancheng Bioengineering Institute (Nanjing, China).

### 2.9 Quantitative real-time RT-PCR analysis

The expression of nutrient transport related genes (*GLUT1*, *GLUT2*, *SNAT1*, *SNAT2*, *SNAT4*, *PepT1*, *ANG2*, and *VEGFA*) in placental tissue, intestinal barrier related genes (*MUC1*, *MUC2*, *claudin1*, *occludin*, and *ZO-1*) in jejunal tissue, and inflammation related genes (*IL-1β*, *IL-6*, *IL-8*, *IL-10*, *TNF-α* and *IFN-γ*) in jejunal tissue were detected by q-PCR. The gene primer sequences were shown in Table 3. Tissue samples were lysed using TRIzol and homogenized with a homogenizer. Total RNA was extracted from jejunal mucosa using TRIzol reagent (TaKaRa Biotechnology Co, Ltd., Dalian, China) according to the manufacturer’s instructions. The concentration and purity of RNA was analysed spectrophotometrically (Beckman Coulter DU800; Beckman Coulter Inc.), taking into account the ideal absorbance ratio (1.8 A260/280 2.0). Reverse transcription was performed using the PrimeScript^™^ RT Reagent (TaKaRa, Japan) kit and stored at −20°C for testing. Reference TB Green^™^ Premix Ex Taq^™^ II (TaKaRa, Japan) instruction manual was used with QuantStudio5 (Applied Biosystem) detection system for real-time fluorescence quantitative PCR. The cycling conditions were as follows predenaturation at 95°C for 30 s and 40 cycles of denaturation at 95°C for 5 s, annealing for 30 s and extension at 60°C for 34 s.

**Table 3:** Primer sequences of the target genes.

Gene	Primer sequences (5′–3′)	Accession No.
*MUC1*	F: GTGCCGCTGCCCACAACCTG	XM_001926883.5
	R: AGCCGGGTACCCCAGACCCA	
*GLUT1*	F: GCCTGAGACCAGTTGAAAGCAC	XM_021096908.1
	R: CTGCTTAGGTAAAGTTACAGGAG	
*GLUT3*	F: TGCACGGGCTTTGTGCCGATG	XM_021092391.1
	R: AAGGAGGTGAAGATTAGGAA	
*SNAT1*	F: AAGAACCTGGGCTATCTCGG	XM_003355629
	R: TGTTGCGTTAGGACTCGTTG	
*SNAT2*	F: GTTACCTTTGGTGATCCAGGC	NM_018976
	R: ACCAATGACACCAGCAGAACC	
*SNAT4*	F: GTTCTTTGCCTTCACTCACTA	XM_003355630
	R: GACCCAAGCCTCCAGATT	
*ANG2*	R: AATAACTGTCCATCCACCTCCA	NM_213808.1
	F: AGCCTTCAGGAACAAGAGTGC	
*VEGFA*	F: GAACTTTCTGCTCTCTTGGGT	NM_214084.1
	R: GGTTTCTGGTCTCCTTCTGCC	
*GLUT2*	F: GACACGTTTTGGGTGTTCCG	NM_001097417.1
	R: GAGGCTAGCAGATGCCGTAG	
*PepT1*	F: GCCAAAGTCGTCAAGTGC	NM_214347
	R: GGTCAAACAAAGCCCAGA	
*IL-1β*	F: CGTGCAATGATGACTTTGTCTGT	NM_214055.1
	R: AGAGCCTTCAGCATGTGTGG	
*IL-6*	F: TTCACCTCTCCGGACAAAAC	NM001252429.1
	R: TCTGCCAGTACCTCCTTGCT	
*IL-8*	F: AGTTTTCCTGCTTTCTGCAGCT	NM213867.1
	R: TGGCATCGAAGTTCTGCACT	
*IL-10*	F: TAATGCCGAAGGCAGAGAGT	NM_214041.1
	R: GGCCTTGCTCTTGTTTTCAC	
*TNF-α*	F: CGTGAAGCTGAAAGACAACCAG	NM_214022.1
	R: GATGGTGTGAGTGAGGAAAACG	
*IFN-λ*	F: TGCATCACATCCACGTCGAA	NM001142837.1
	R: GCAGCCTTGGGACTCTTTCT	
*MUC2*	F: GGTCATGCTGGAGCTGGACAGT	XM021082584.1
	R: TGCCTCCTCGGGGTCGTCAC	
*Claudin-1*	F: GCATCATTTCCTCCCTGTT	NM001161638.1
	R: CATGACTTCTGCCCTGACGA	
*Occludin*	F: AACTTCCACTGATGTCCCCCGT	NM001163647.2
	R: CCTAGACTTTCCTGCTCTGCCC	
*ZO-1*	F: CGTGTCAACGCCACTATCA	XM021098896.1
	R: TTGTCTTCCAAAGCCCCT	
*β-actin*	F: GGATGACGATATTGCTGCGC	XM003124280.5
	R: GATGCCTCTCTTGCTCTGGG	

ANG, angiogenin; GLUT, glucose transport protein; IFN-*λ*, interferon-*λ*; IL-10, interleukin-10; IL-1β, interleukin-1β; IL-6, interleukin-6; IL-8, interleukin-8; MUC, mucin; PepT1, oligopeptide transporter 1; SNAT, basolateral transporters system A; TNF-α, tumor necrosis factor; VEGFA, vascular endothelial growth factor; ZO-1, zona occludens-1.

### 2.10 Western blotting

The western blot (WB) method was used to detect the expression of claudin1 and occludin protein in jejunal tissue. Jejunal tissue samples were mechanically and ultrasonically crushed, centrifuged, and the supernatant was collected for determination of total protein concentration using the BCA protein assay (Beyotime, Beijing, China). Equal amounts of protein were separated on 10% SDSPAGE gels, and then electrically transferred to polyvinylidene difluoride (PVDF) membranes. The PVDF membranes were blocked with 5% skimmed milk powder for 1 h at room temperature. Subsequently, they were incubated with the different primary antibody [Abcam (Cambridge, MA, United States)] at 4 degrees for 12 h. Then the PVDF membranes were incubated with corresponding secondary antibodies [Santa Cruz Biotechnology (Santa Cruz, CA, United States)] for 1 h at room temperature, followed by testing the target protein bands using ECL Luminescence Detection Kit (Beyotime, Beijing, China). Image Lab 5.1 was used for exposure photography and strip grayscale value analysis. Antibody concentration of claudin1, occludin, β-actin are 1:1,000.

### 2.11 Statistical analysis

Statistical differences were analyzed by the independent samples *t*-test in SPSS 22.0 statistics software (Chicago, IL, United States). Independent sample *t*-tests were performed for pairwise comparisons between NBW, IUGR, and IUGR + COS. Firstly, the comparison between NBW and IUGR aimed to verify the successful establishment of the IUGR piglet injury model. Secondly, the analysis between IUGR and IUGR + COS was conducted to evaluate the improvement effect of COS supplementation on IUGR piglets. Lastly, the comparison between NBW and IUGR + COS assessed the remaining gap, if any, between IUGR piglets after COS supplementation and normal piglets. The results were expressed as mean ± standard error, with significance indicated by *p* < 0.05 and a trend indicated by 0.05 ≤ *p* < 0.10.

## 3 Results

### 3.1 Sows’ reproductive performance

The results of sows’ reproductive performance were shown in Table 4. The supplementation of COS in sow diet had no significant effect on the total born, born alive, mean BW and mean litter weight at birth, litter weight and average pig weight at weaning, as well as average daily gain of piglets during lactation (*p* > 0.05), but significantly reduced the number of stillbirths and mummies (*p* < 0.05). Furthermore, COS group exhibited a 35.22% reduction in the number of piglets weighing less than 1 kg compared to the control group. After supplementing with COS, the incidence of IUGR decreased by 8.57% compared to the control group.

**Table 4:** Effects of maternal chitosan oligosaccharides supplementation during late gestation and lactation on the reproductive performance of sows.

Item	Control	COS	*p*-value
Birth, piglets/litter, *n* = 15			
Total born, *n*	12.47 ± 0.71	12.53 ± 0.56	0.94
Born alive, *n*	11.00 ± 0.49	11.93 ± 0.65	0.26
Born dead, *n*	0.93 ± 0.18	0.40 ± 0.13	0.02
Stillborn, *n*	0.60 ± 0.19	0.13 ± 0.09	0.04
BW <1.0 kg, *n*	2.47 ± 0.64	1.60 ± 0.49	0.29
Percentage of IUGR, %	20.59 ± 6.43	12.02 ± 3.71	0.26
Mean BW at birth, kg	1.34 ± 0.09	1.40 ± 0.06	0.63
Mean litter weight at birth, kg	14.37 ± 0.86	16.42 ± 1.03	0.14
Weaning, *n* = 10			
Litter size after cross-foster, *n*/litter	10.80 ± 0.13	10.70 ± 0.26	0.74
Litter size at weaning, piglets/litter	9.00 ± 0.30	9.10 ± 0.23	0.80
Litter weight after cross-foster, kg	16.02 ± 0.95	14.85 ± 0.55	0.30
Litter weight at weaning, kg	52.37 ± 2.61	54.89 ± 2.61	0.50
Average pig weight at weaning, kg	5.84 ± 0.27	6.03 ± 0.13	0.53
ADG for piglet, g	207.37 ± 13.21	220.96 ± 6.17	0.37
Lactation ADFI for sow, kg	5.05 ± 0.21	5.13 ± 0.22	0.79
Sow backfat thickness, mm			
Day 85 of gestation (*n* = 15)	13.64 ± 0.72	14.00 ± 0.47	0.68
Farrowing (*n* = 10)	14.27 ± 0.69	14.90 ± 0.53	0.49
Weaning (*n* = 10)	12.64 ± 0.49	13.80 ± 0.61	0.15
Decrease in backfat thickness during lactation (*n* = 10)	1.64 ± 0.34	1.10 ± 0.23	0.22

### 3.2 Placenta function-related genes expression of sows

**Figure 1:**
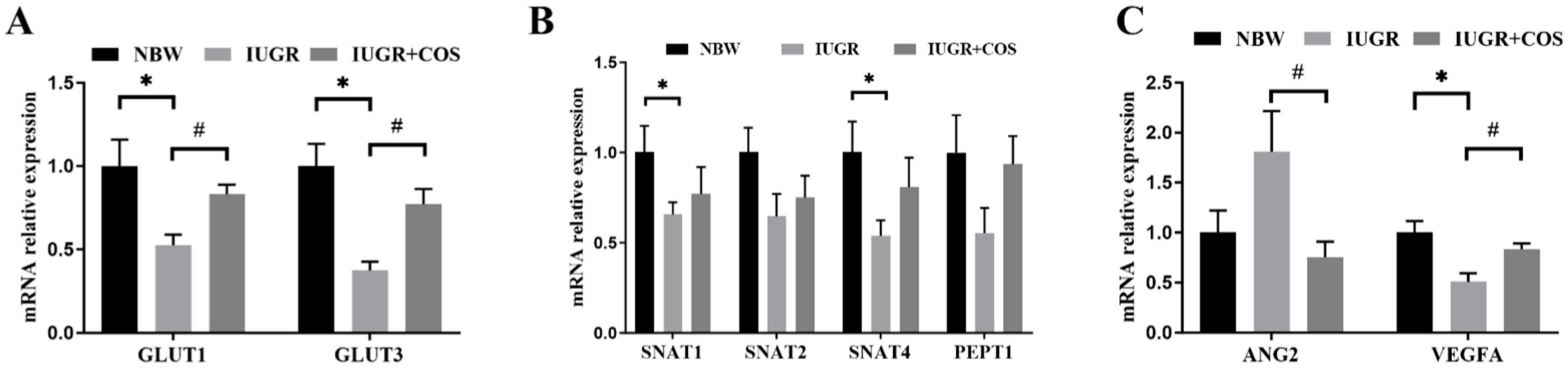
Effects of maternal chitosan oligosaccharides supplementation during late gestation on the placenta function-related genes expression of sows. (A) Glucose transporters: *GLUT1* and *GLUT3*. (B) Amino acid transporters: *SNAT1*, *SNAT2*, *SNAT4*, and *PepT1*. (C) Angiogenesis: *ANG2* and *VEGFA*. ^*^Means significant difference between NBW vs. IUGR/IUGR + COS. ^#^Means significant difference between IUGR vs. IUGR + COS, *p* < 0.05, *n* = 10.

As shown in Figure 1, the mRNA expression of glucose transport related genes (*GLUT1* and *GLUT3*), amino acid transport related genes (*SNAT1* and *SNAT4*), and angiogenesis gene *VEGFA* in the placenta of IUGR piglets (from control sows) were significantly lower than those in the placenta of normal piglets (from control sows) (*p* < 0.05); However, the expression of *GLUT1*, *GLUT3* and *VEGFA* mRNA in the placenta of IUGR piglets (from COS sows) was significantly higher than that in the placenta of IUGR piglets (from control sows) (*p* < 0.05). The expression of *ANG2* mRNA in the placenta of IUGR piglets (from COS sows) was significantly lower than that in the placenta of IUGR piglets (from control sows) (*p* < 0.05).

### 3.3 Placental antioxidant status and cytokine of sows

As shown in Table 5, the levels of ATP, GSH, IL-10 and GSH/GSSG in the placenta of IUGR piglets (from control sows) were significantly lower than those in the placenta of normal body weight piglets (from control sows), whereas the concentrations of MDA and GSSG exhibited an opposite trend (*p* < 0.05). Furthermore, maternal dietary COS supplementation resulted in a significant decrease in placental MDA levels in IUGR piglets, while there was a significant increase in GSH levels (*p* < 0.05). Compared to normal sow placenta (NBW), the levels of ATP, GSH, GSH/GSSG, and IL-10 remained significantly lower (*p* < 0.05), whereas MDA was significantly higher (*p* < 0.05) after COS supplementation of sow diets (IUGR + COS).

**Table 5:** Effects of maternal chitosan oligosaccharides supplementation during late gestation on the placenta oxidative parameters and cytokines levels of sows.

Item	Control		COS	*p*-value		
	NBW	IUGR	IUGR	*p*1	*p*2	*p*3
ATP, nmol/mg prot	483.28 ± 61.76	239.82 ± 18.76	236.47 ± 50.80	0.01	0.95	0.01
MDA, μmol/mg prot	3.66 ± 0.17	5.01 ± 0.15	4.28 ± 0.12	<0.01	0.01	0.01
GSH, μmol/mg prot	2.52 ± 0.20	1.30 ± 0.07	1.70 ± 0.12	<0.01	0.01	<0.01
GSSG, μmol/mg prot	9.53 ± 1.76	16.54 ± 2.71	12.96 ± 1.45	0.04	0.26	0.15
GSH/GSSG	0.40 ± 0.09	0.12 ± 0.03	0.15 ± 0.02	0.02	0.47	0.02
IL-1β, ng/mg prot	14.56 ± 0.90	14.77 ± 1.40	13.77 ± 0.94	0.90	0.56	0.55
IL-10, ng/mg prot	107.21 ± 4.98	71.73 ± 7.64	76.21 ± 6.82	<0.01	0.67	0.02
TNF-α, pg./mg prot	129.16 ± 6.63	135.75 ± 11.61	135.28 ± 9.19	0.63	0.98	0.60

*p*-value means significant difference between two groups, *p*1: NBW vs. IUGR, *p*2: IUGR vs. IUGR + COS, *p*3: NBW vs. IUGR + COS, *n* = 10. MDA, malondialdehyde; GSH, glutathione; GSSG, oxidized glutathione; IL-1β, interleukin-1β; IL-10, interleukin-10; TNF-α, tumor necrosis factor-α.

### 3.4 Jejunal digestive enzyme activities of 21-day-old suckling piglets

**Figure 2:**
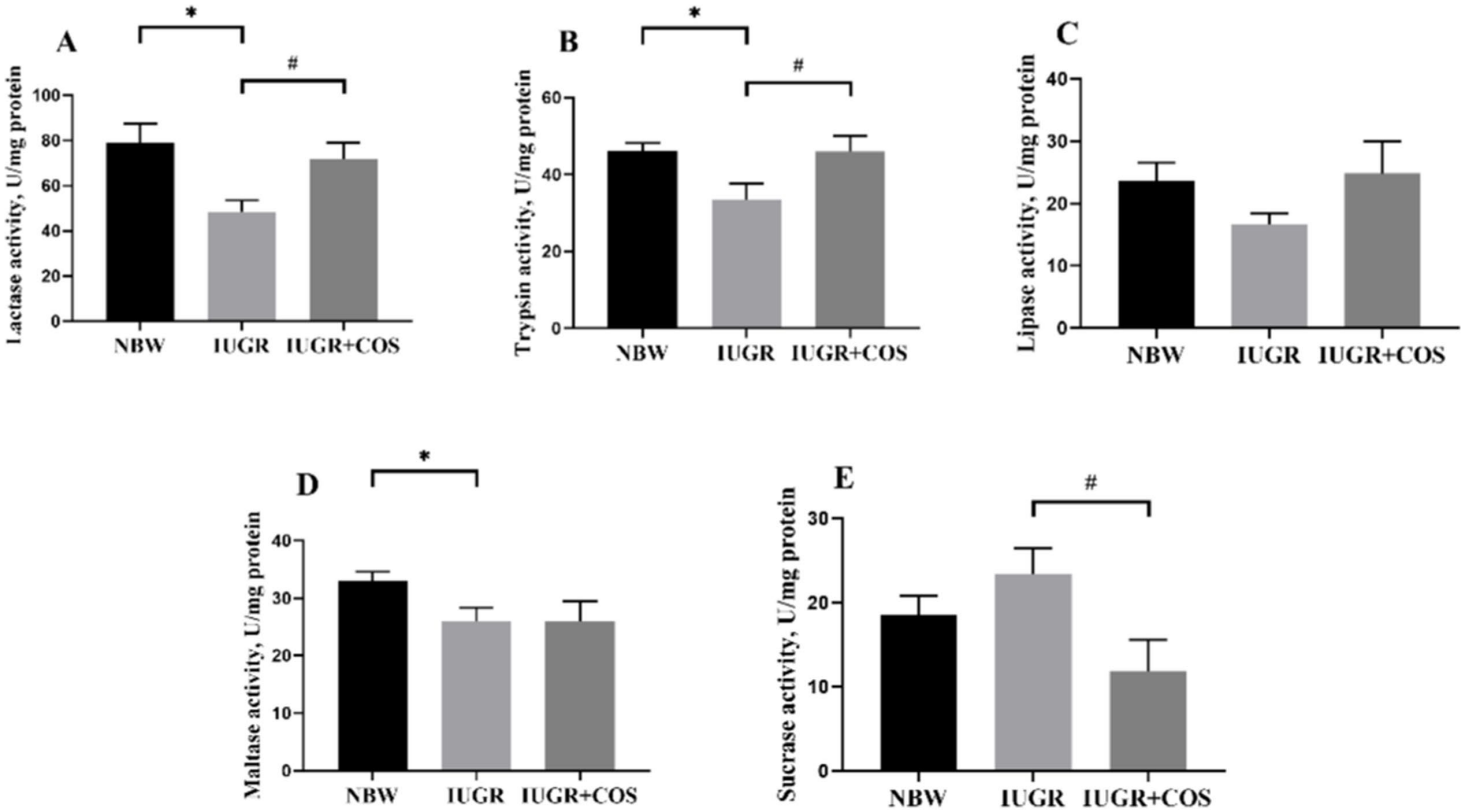
Effects of maternal chitosan oligosaccharides supplementation during late gestation and lactation on the jejunal digestive enzyme activities of 21-day-old suckling piglets. **(A)** lactase activity; **(B)** trypsin activity; **(C)** lipase activity; **(D)** maltase activity; **(E)** sucrase activity. ^*^Means significant difference between NBW vs IUGR/IUGR + COS, ^#^Means significant difference between IUGR vs IUGR + COS, *p* < 0.05, *n* = 8.

As shown in Figure 2, the activities of jejunal trypsin, lactase and maltase in 21-day-old IUGR piglets were significantly lower compared to those of normal body weight piglets (*p* < 0.05). Maternal dietary COS supplementation during late pregnancy and lactation significantly increased the activities of jejunal trypsin and lactase in 21-day-old IUGR piglets (*p* < 0.05), and significantly reduced sucrase activity (*p* < 0.05).

### 3.5 Jejunal barrier function of 21-day-old suckling piglets

**Figure 3:**
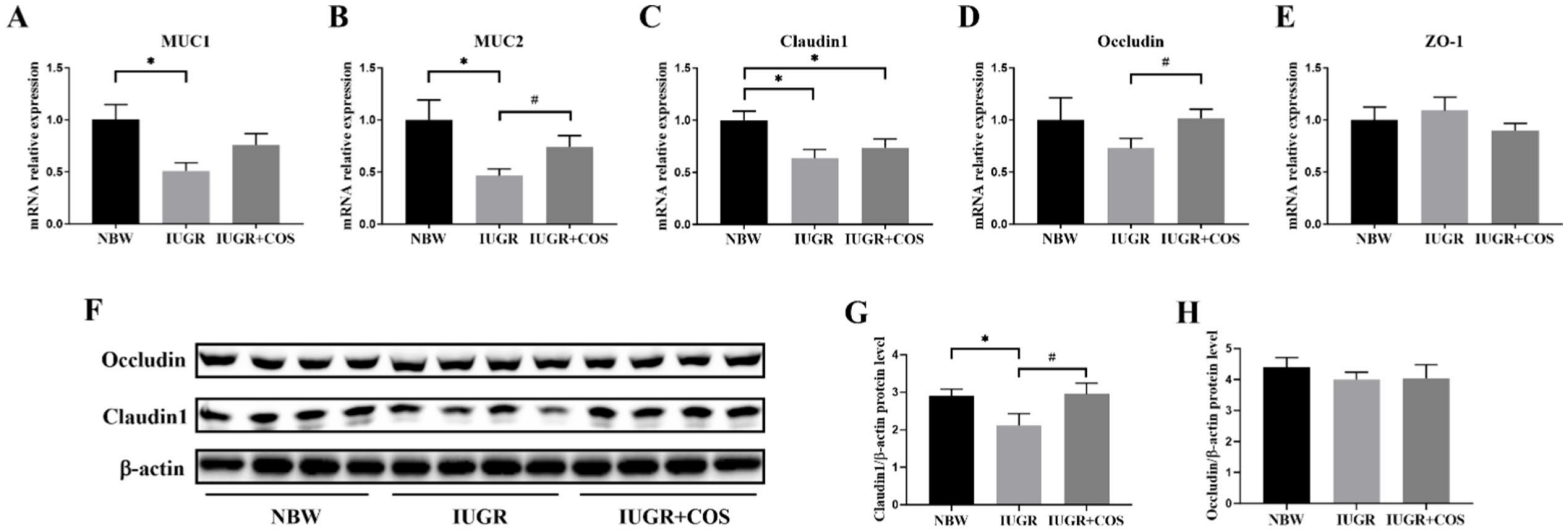
Effects of maternal chitosan oligosaccharides supplementation during late gestation and lactation on the jejunal barrier function of 21-day-old suckling piglets. (A–E) Jejunal barrier-related gene expression levels, *n* = 8. (F–H) Jejunal barrier-related protein expression levels, *n* = 4. ^*^Means significant difference between NBW vs. IUGR/IUGR + COS. ^#^Means significant difference between IUGR vs. IUGR + COS, *p* < 0.05.

As shown in Figure 3, compared with normal body weight piglets, the expression of *MUC1*, *MUC2*, *claudin1* mRNA and claudin1 protein in the jejunum of 21-day-old IUGR piglets (from control sows) were significantly reduced (*p* < 0.05). Supplementing COS to the diet of sows during late gestation and lactation resulted in a significant increase in the expression of *MUC2* and *occludin* mRNA as well as claudin1 protein in the jejunum of 21-day-old IUGR piglets (*p* < 0.05).

### 3.6 Gene expression levels of cytokines in the jejunum of 21-day-old suckling piglets

**Figure 4:**
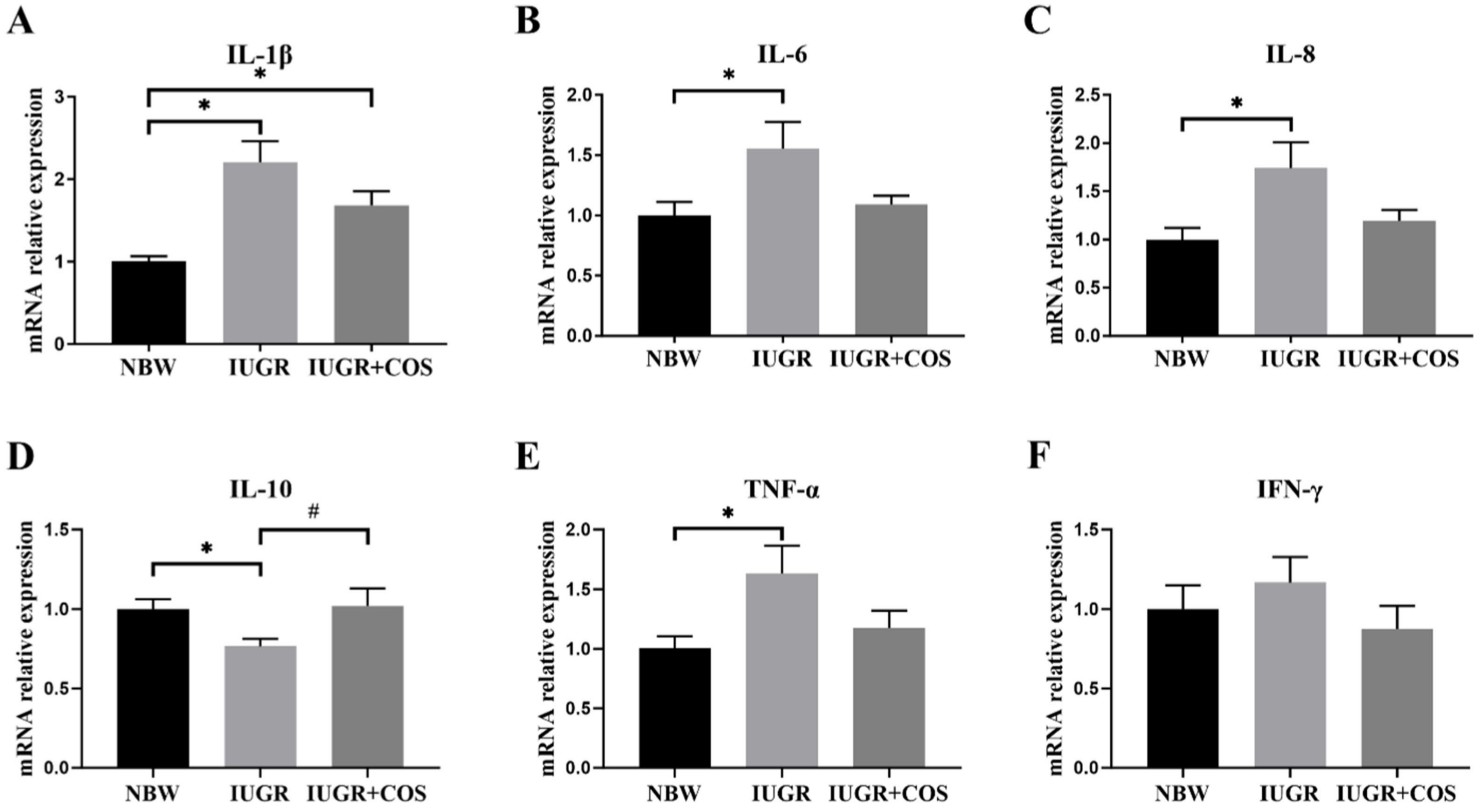
Effects of maternal chitosan oligosaccharides supplementation during late gestation and lactation on the jejunal cytokine gene expression levels of 21-day-old suckling piglets. **(A)** IL-1β (Interleukin-1β); **(B)** IL-6 (Interleukin-6); **(C)** IL-8 (Interleukin-8); **(D)** IL-10 (Interleukin-10); **(E)** TNF-α (Tumor necrosis factor-α); **(F)** IFN-γ (Interferon-γ). ^*^Means significant difference between NBW vs IUGR or NBW vs IUGR + COS, ^#^Means significant difference between IUGR vs IUGR + COS (P3), *p* < 0.05, *n* = 8.

As shown in Figure 4, the expression of *IL-1β*, *IL-6*, *IL-8*, and *TNF-α* mRNA in the jejunum of 21-day-old suckling IUGR piglets was significantly increased compared to normal body weight piglets (*p* < 0.05), while the expression of *IL-10* mRNA was significantly reduced (*p* < 0.05). Supplementation of COS to the diet of sows during late pregnancy and lactation resulted in a significant increase in the expression of *IL-10* mRNA in the jejunum of piglets (*p* < 0.05), with a tendency to decrease the expression of *IL-6* (*p* = 0.06) and *IL-8* (*p* = 0.08) mRNA compared to IUGR piglet from control sows.

### 3.7 Antioxidant status of jejunum in 21-day-old suckling piglets

The results of the antioxidant status of jejunum in suckling piglets were presented in Table 6. Compared to 21-day-old normal body weight piglets, IUGR piglets (from control sows) exhibited a significant increase in MDA and GSSG levels (*p* < 0.05), a significant reduction in the GSH/GSSG ratio (*p* < 0.05), and a tendency to increase CAT level (*p* = 0.06). The addition of COS to the diet of sows during late pregnancy and lactation significantly reduced the MDA content in the jejunum of IUGR piglets (*p* < 0.05). However, GSSG remained significantly elevated (*p* < 0.05) and GSH/GSSG was significantly reduced (*p* < 0.05) after COS supplementation of the sows’ diets (IUGR + COS), compared with 21-day-old normal body weight piglets.

**Table 6:** Effects of maternal chitosan oligosaccharides supplementation during late gestation and lactation on the antioxidant status of jejunum in 21-day-old suckling piglets.

Variable	NBW	IUGR	IUGR + COS	*p*1	*p*2	*p*3
MDA, μmol/mg prot	0.98 ± 0.04	1.26 ± 0.05	1.04 ± 0.04	0.01	0.01	0.28
CAT, U/mg prot	6.13 ± 0.47	9.51 ± 1.51	7.16 ± 0.46	0.06	0.17	0.14
T-AOC, U/mg prot	282.00 ± 37.91	255.31 ± 32.64	275.66 ± 48.95	0.60	0.73	0.92
GSH-PX, U/mg prot	12.57 ± 1.28	11.21 ± 1.88	13.04 ± 2.32	0.56	0.55	0.86
GSH, μmol/mg prot	1.07 ± 0.06	1.01 ± 0.04	1.06 ± 0.09	0.43	0.64	0.90
GSSG, μmol/mg prot	9.09 ± 0.72	14.01 ± 1.54	13.47 ± 1.18	0.01	0.78	0.01
GSH/GSSG	0.13 ± 0.02	0.08 ± 0.01	0.08 ± 0.01	0.01	0.54	0.03

*p*-value means significant difference between two groups, *p*1: NBW vs. IUGR, *p*2: IUGR vs. IUGR + COS, *p*3: NBW vs. IUGR + COS, *n* = 8. MDA, malondialdehyde; CAT, catalase; T-AOC, total antioxidant capacity; GSH-PX, glutathione peroxidase; GSH, glutathione; GSSG, oxidized glutathione.

## 4 Discussion

The main purpose of this study is to investigate the effect of supplementing COS to the diet of sows during late gestation and lactation on placental function, as well as its effect on intestinal function of IUGR piglets after 21 days of suckling. Previous research has utilized various methods, such as direct nutrient supplementation to piglets post-birth, in order to improve the growth performance and functions of IUGR (19, 20). From another perspective, supplementing nutrients during late gestation and lactation in sows has been demonstrated to improve the reproductive performance of sows and the growth performance of piglets (21, 22). In previous studies, maternal dietary COS supplementation during late gestation and lactation has been shown to significantly increase the average daily gain and weaning weight of piglets, but had no significant effect on the birth weight (16). In addition, it can also improve the born alive (14). Other studies have shown that the supplementation of COS can increase the total number of newborn piglets and live piglets, as well as significantly improve the average live litter weight, however, it has no significant effect on the average daily gain and average weaning weight (18). In this experiment, we found that dietary COS supplementation in sows only reduced the occurrence of stillbirths and mummies, without significant effects on other productive parameters. The results of this study were partially inconsistent with previous findings, possibly due to individual variations among sows and environmental conditions. Additionally, differences in sow management and nutritional control during gestation may also influence reproductive performance. Nevertheless, it is worth noting that our study found a 35.22% reduction in piglets with a birth weight less than 1 kg in the COS group compared to the control group, indicating potential alleviation of IUGR occurrence through maternal COS supplementation during late gestation.

The placenta serves as a crucial interface between the maternal and fetal compartments. It plays an essential role in ensuring the successful progression of pregnancy and the optimal growth and development of the fetus. During gestation, the placenta is responsible for transporting nutrients. Dysregulated nutrient transport can result in intrauterine growth restriction (23). Our Findings indicating impaired placental nutrient transport as a contributing factor to IUGR, COS supplementation during late gestation effectively ameliorated decreased nutrient transport function following placental injury. Previous studies have emphasized the importance of adequate vascularization within the placenta for normal fetal development (24), however, IUGR placentas exhibited significantly poorer vascular development compared to normal placentas (8). In this study, it was found that impaired vascular growth in sow placenta appeared to be another factor contributing to the emergence of IUGR. However, this impaired state could be restored to a certain extent after COS supplementation. It is important to note that placental nutrient transport and vascular development are interdependent, compromised vasculature limits exchange capacity in IUGR placentae (23). The above results indicated that supplementing sows feed with COS during late gestation effectively enhanced placental nutrient transport capacity, improved angiogenesis within the placenta, and reduced the occurrence of IUGR piglets.

Due to the close connection between the mother and the fetus, facilitated by the placenta as a conduit, maternal well-being significantly influences fetal development. In late pregnancy in sows, activities such as fetal growth, mammary gland development, and milk production resulted in elevated levels of reactive oxygen species (ROS), leading to oxidative stress in sows (25). Maternal oxidative stress is associated with an increased risk of IUGR (25). The placenta, an extremely important organ in this process, is highly sensitive to oxidative stress (26). Our study indicating pronounced oxidation in IUGR sows’ placentas and confirming the link between IUGR and oxidative stress. Following COS treatment, effective alleviation of placental oxidative stress. Furthermore, there are reports suggesting that immune cells undergo functional alterations during late gestation, resulting in increased release of inflammatory cytokines which can affect the maternal-fetal health. In this study, we found the same situation in the placenta. This increase in pro-inflammatory cytokines coupled with a decrease in anti-inflammatory cytokines reflects immune damage and inflammation within sow placentas during pregnancy, potentially contributing to adverse outcomes such as IUGR (27). However, following COS supplementation, the inflammatory response was alleviated, to some extent compared to IUGR. Therefore, supplementing COS can effectively alleviate oxidative stress and inflammatory response in the placenta, ultimately reducing the emergence of IUGR piglets.

After parturition, the primary connection between the neonate and the dam is through colostrum and milk, which plays an essential part in neonatal intestinal health. Piglets primarily acquire immunoglobulins by sucking colostrum postnatally, however, IUGR piglets may experience inadequate sucking or exposure to foodborne pathogens due to developmental impairment, resulting in further intestinal damage (6). In this study, changes in jejunal digestive enzyme activity in piglets reflected the addition of COS can effectively improve digestive and absorption function in piglets’ intestines.

The intestinal health of piglets can impact their overall growth, and inadequate development in the mother’s body and insufficient acquisition of colostrum and milk during lactation can have a detrimental effect on the intestinal health of IUGR piglets. This study observed impaired gut barrier, inflammatory response, and oxidative stress in IUGR piglets. Supplementing COS to the diet of sows during lactation can significantly ameliorates intestinal damage in IUGR piglets. Additionally, maternal COS also alleviated the inflammatory response in IUGR suckling piglets. We found that lipid peroxidation occurred in IUGR piglets’ bodies and a change in cellular redox state. However, supplementation of COS to lactating sow diets can inhibit lipid peroxidation. Previous studies have confirmed that supplementing COS to piglet feed can significantly improve the antioxidant defense system function of weaned piglets, protect them from oxidative stress, and improve their immune status (9). In this study, by supplementing COS to lactating sows feed resulted in the transmission of these effect to the piglets, possibly due to some antioxidant and anti-inflammatory components present in mother’s milk, thereby enhancing piglet antioxidant and immune status.

This study primarily focuses on improving IUGR in piglets by supplementing sows with COS through the placenta. By enhancing placental angiogenesis and nutrient transport, we aim to promote healthier fetal development and reduce IUGR incidence. Although this study did not examine the composition and changes of colostrum and regular milk, COS supplementation in sows may help alleviate intestinal damage in IUGR piglets via breast milk. However, research on COS’s impact on milk is limited, and it remains uncertain whether its anti-inflammatory and antioxidant properties extend to milk.

Furthermore, the role of microorganisms cannot be overlooked. After birth, close contact between piglets and their mothers, particularly their initial exposure to sow feces, facilitates the colonization of microorganisms in the piglets’ intestines (28). Consequently, the sow’s diet can also influence the microbial composition of piglets (29). Studies indicate that COS supplementation positively affects host intestinal health and gut microbiota (30). We hypothesize that by improving the sow’s gut microbiota through COS supplementation, beneficial microorganisms can be transmitted to piglets upon contact, thereby enhancing their intestinal health and mitigating the effects of IUGR.

## 5 Conclusion

In this study, the results demonstrated that supplementing COS in the late gestation of sows can improve reproductive performance, and placental, alleviate oxidative stress in the placenta, and consequently reduce the incidence of IUGR piglets. Continuing to supplement COS during lactation can improve the intestinal barrier function of 21-day-old suckling IUGR piglets, alleviate inflammatory reactions and oxidative stress, thus effectively alleviate the adverse effects of IUGR piglets. In conclusion, supplementing sow feed with COS during late gestation and lactation can effectively reduce the incidence of IUGR piglets and improve their health. This experiment also provided nutritional strategies for pregnant and lactating women to enhance infant health.

## Data Availability

The original contributions presented in the study are included in the article/supplementary material, further inquiries can be directed to the corresponding author.
